# Cascaded metalenses boost applications in near-eye display

**DOI:** 10.1038/s41377-024-01699-5

**Published:** 2025-01-20

**Authors:** Jiacheng Sun, Tao Li

**Affiliations:** National Laboratory of Solid State Microstructures, College of Engineering and Applied Sciences, Nanjing University, 210093 Nanjing, China

**Keywords:** Imaging and sensing, Displays, Nanophotonics and plasmonics

## Abstract

Recent advancements show the potential of cascaded metalenses in near-eye display applications, achieving performance that rivals traditional eyepiece systems. By leveraging the human pupil as an aperture and taking into account practical factors such as eye relief, pupil size, and display dimensions, this approach suggests a bright future for the incorporation of meta-optics in cutting-edge near-eye display technologies.

As computing and artificial intelligence progress, technologies like augmented reality (AR) and virtual reality (VR) are transferring those fancy ideas to practical applications^1–3^. A key element of AR and VR systems is the increasing need for near-eye displays. However, traditional near-eye displays are always positioned too close to the eye, making them uncomfortable for extended use^4,5^. To mitigate this issue, optical elements are necessary to adjust the light path and reduce eye strain and discomfort. Unfortunately, conventional refractive optical components tend to be large and bulky, while head-mounted devices require more compact optics, which constrains design possibilities. To tackle this, various compact solutions, such as pancake optics^6,7^, holography^8^, and leaky waveguides^9^, have been proposed. Despite their potential, these approaches encounter issues like low efficiency. Consequently, achieving a balance between portability and high performance continues to be a significant challenge.

Metalenses, which are made of nano-structures arranged in two dimensions, present an innovative approach for compact near-eye display systems. Their ultra-thin and lightweight characteristics render them particularly well-suited for head-mounted devices. With advancements in nano-fabrication technology, significant developments have been reported regarding metalenses operating in the near-infrared and visible spectrum, enabling a variety of applications, including holography^10^, beam shaping^11^, and microscopy^12,13^, and showing considerable promise for AR and VR uses. Nonetheless, achieving a wide field of view (FOV) that mirrors human vision (approximately 120°) is a challenge due to pronounced off-axis aberrations in metalenses. In pioneering designs from several years ago, researchers stacked multiple metalenses into doublet or triplet configurations, allowing for greater design flexibility and enhanced performance, resulting in wide FOV imaging^14,15^. Other strategies involve the combination of single-layer metalenses with apertures, which can produce nearly diffraction-limited imaging across nearly 180° of FOV^16^. Despite their remarkable capabilities, these designs typically feature entrance apertures of less than 1 mm in diameter, which restricts their applicability in practical near-eye display scenarios.

For near-eye displays, it is essential for optical modulation eyepieces to match the size of human eyes, a requirement that surpasses the capabilities of most existing metalens technologies. Unfortunately, the monochromatic Seidel aberrations scale with both aperture size and field angle^17^, creating difficulties for optical devices to maintain high quality while also possessing large apertures and expansive fields of view. To address this challenge, recent advancements in machine learning and algorithm optimization present an effective and practical solution. Consequently, multi-layer metalenses have emerged as a promising method for achieving both a wide FOV and a large aperture in light field modulations.

In a recently published paper in *Light: Science & Applications*, a team led by Prof. Arka Majumdar from the University of Washington, along with Prof. Juejun Hu and Prof. Tian Gu from MIT, has introduced a metalens doublet system with a large aperture, specifically designed for near-eye displays^18^. This innovative configuration, which incorporates two-layer metalenses and a pupil, achieves a broad field of view (FOV) of up to 60° and even 80°. Unlike traditional metalens doublet cameras, this system is designed to project images far enough from the eyes, enabling users to comfortably observe an expansive scene that appears to be at infinity, despite the display being situated close to the eyes. Figure 1 illustrates the working principle of this system. In Fig. 1a, it is evident that the optimal viewing distance for the human eye exceeds several decimeters, while a head-mounted near-eye display is positioned too closely for comfort. As shown in Fig. 1b through ray-tracing simulations, the near-eye display can simulate a distant scene by utilizing the human pupil as an aperture.

**Fig. 1:**
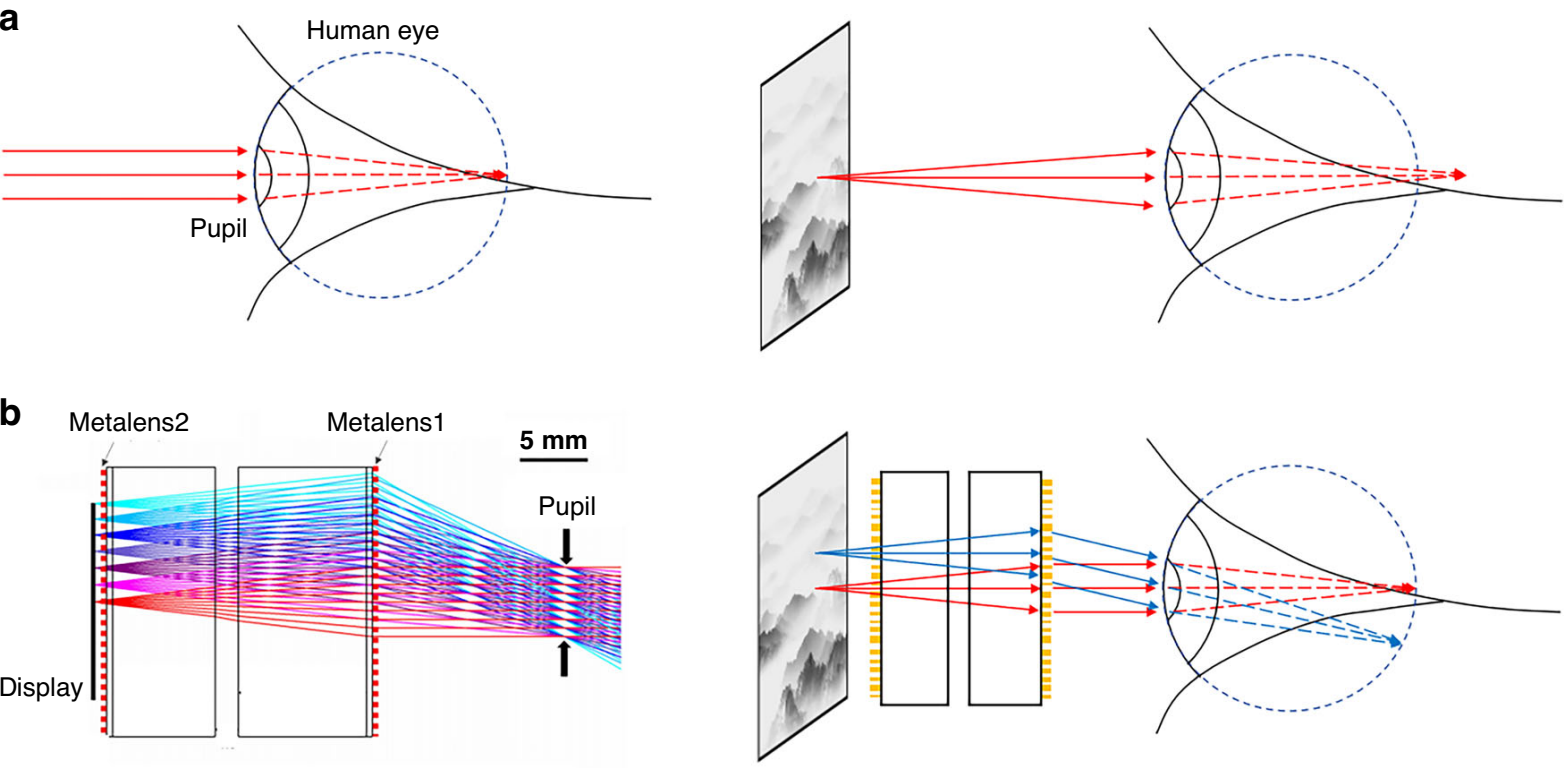
Illustration of working principles of human eyes and the metalens doublet. **a** The distance from the head-mounted near-eye display to the eyes is too close to make people comfortable. **b** The double-layer metalens simulate a near-eye display as an infinite distance scene through the human pupil

Utilizing the human pupil as a diaphragm is a remarkable innovation. Instead of merely projecting an image onto a sensor, this approach enhances direct and natural vision. To test this concept, researchers developed and optimized two metalens doublets with diameters in the centimeter range, paired with apertures measuring several millimeters, effectively mimicking the dimensions of the human eye and pupil, respectively. They then constructed an eye model that included an iris (serving as the pupil), a refractive lens (representing the eye’s lens), and a camera sensor, which allowed them to achieve high-quality imaging at angles of incidence up to 30°, corresponding to a 60° of full FOV. This successfully demonstrated the doublet’s effectiveness for near-eye displays. To be mentioned, the imaging quality could be further enhanced through better device alignment, optimized filters, and the use of non-air spacers with suitable refractive indices, which are all achievable in industrial production^19^.

This work demonstrates the remarkable capability of metalenses in near-eye display applications. By optimizing the compact design, aperture size, and FOV, the performance of a metalens doublet has been achieved that rivals that of conventional refractive eyepieces, meeting common benchmarks for near-eye display. Although the imaging performance near the diffraction limit cannot be sustained throughout the entire FOV, the requirements for ultra-wide FOV in applications like AR/VR or night vision are inherently relaxed due to the nature of human eye fixation points^20^. This advancement highlights the significant role of meta-optics that could play in advancing high-performance near-eye displays and night vision systems.
